# Indian scorpions collected in Karnataka: maintenance in captivity, venom extraction and toxicity studies

**DOI:** 10.1186/s40409-015-0053-4

**Published:** 2015-12-04

**Authors:** Santhosh Kambaiah Nagaraj, Pavana Dattatreya, Thippeswamy Nayaka Boramuthi

**Affiliations:** Department of Post-graduate Studies and Research in Microbiology, Jnana Sahyadri campus, Kuvempu University, Shivamogga, Karnataka India

**Keywords:** Scorpion venom, Restrainer, *Hottentotta*, *Heterometrus*, Venom extraction, LD_50_

## Abstract

**Background:**

Maintenance of scorpions under laboratory conditions is ideal for long-term venom collection to explore the therapeutic applications of scorpion venom. Collection of venom by electrical stimulation requires a reliable stimulator and effective restrainer. Thus, the present study was conducted to develop a convenient method to maintain scorpions and to extract their venom for toxicity studies via a modified restrainer and stimulator.

**Methods:**

Four different scorpion species were collected, among which three species were maintained in the laboratory in containers that mimic their natural habitat. Venom was extracted from *Hottentotta rugiscutis* by electrical stimulation at 8 V for 18 months and LD_50_ was estimated by the graphic method of Miller and Tainter.

**Results:**

A total of 373 scorpions including *Hottentotta rugiscutis*, *Hottentotta tamulus, Lychas tricarinatus* and *Heterometrus swammerdami* were collected, identified and maintained successfully, achieving a 97 % survival rate. *Hottentotta rugiscutis* yielded 6.0 mL of venom by electrical stimulation. The LD_50_ of *H. rugiscutis* venom was estimated to be 3.02 mg/kg of body weight in female Swiss albino mice.

**Conclusions:**

Scorpions were successfully maintained for 18 months. Herein we have also documented a simple, cost-effective method of venom extraction by electrical stimulation using a modified restrainer. Furthermore, *Hottentotta rugiscutis* was reported for the first time in Karnataka.

## Background

Scorpions are arthropods belonging to the class Arachnida and order Scorpiones. Globally, 1988 species are known, among which 113 species from 25 genera belonging to six families have been recorded in India [1]. Scorpions of the family Buthidae are of prime importance due to the lethality of their venom to humans, especially to children in many tropical regions [2]. A Buthidae family scorpion, *Hottentotta tamulus* (Fabricius, 1798), is widely distributed throughout the Deccan plateau of southern India [3, 4].

As shown by an internal survey, *Hottentotta rugiscutis* (Pocock, 1897) scorpions are prevalent, live alongside the human habitat in the Chirathagundu village (Karnataka, India) and are known to cause considerable public health problems due to envenomations compared to three other regions – namely Hiriyuru, Nandihalli and Hindaskatte – where human activity is limited. As there is hardly any data on the lethality of *H. rugiscutis* venom, it is important to research these scorpions and to find out their effects on the local population.

Scorpion venom is a complex mixture of mucopolysaccharides, hyaluronidase, phospholipase, serotonin, histamine, enzyme inhibitors, and toxins, mainly neurotoxins that affect the function of Na^+^, K^+^ and other ion channels – and is used as investigatory tool in physiological and pharmacological research. The therapeutic properties of scorpion venom includes anticancer, antimicrobial, antiepileptic, analgesic, antimalarial, pesticidal and insecticidal activities and also may be used in modulating cardiovascular effects and autoimmune diseases [5].

To study the biological importance of venom and for the preparation of antivenom to treat human envenomations, a substantial amount of venom is needed and can be obtained by in-field venom collection or from scorpions maintained under laboratory conditions. In comparison with the former, the latter ensures that copious venom can be extracted at frequent intervals for a longer time. Long-term extraction or milking of venom from a pool of scorpions requires an efficient maintenance of scorpions, which otherwise would exhibit a difference in both the quantity and quality of venom obtained. Furthermore, to date, not much information is available on the effective maintenance of scorpions under laboratory conditions.

There are three main methods for the extraction of venom, namely maceration of the telson, manual stimulation and electrical stimulation of scorpions. Maceration involves the snipping and crushing of the telson to access the venom. It is a reliable method but has the obvious drawback of allowing only one extraction per individual animal. In the manual stimulation method, the animal is provoked manually to secrete the venom on a piece of parafilm [6]. But disadvantages include difficulty in provoking the scorpions [7], low venom yield and less toxicity as compared to the venom obtained by the electrical stimulation method [7–9]. In the third method, stimulation via mild electric shock results in contraction of muscles around the telson causing the venom to squeeze out of the vesicle. However, extracting the venom by this method requires not only a cost-effective and reliable stimulator but also a restrainer.

Given this context, the present study was conducted to develop a convenient method for scorpion maintenance and venom extraction for toxicity studies with the aid of a modified restrainer and stimulator.

## Methods

### Animals

All the experiments were performed according to the guidelines approved by the Institutional Animal Ethics Committee (National College of Pharmacy, Shivamogga, Karnataka; no: NCP/IAEC/CL/206/01). Five groups of female Swiss albino mice aged two months (weighing 20–25 g), with each group containing five mice, were used for testing each dose of venom for toxicity and a sixth group was used as control. The mice were kept under room temperature where they had *ad libitum* access to rodent chow and tap water throughout the experiment.

### Collection of scorpions

**Fig. 1:**
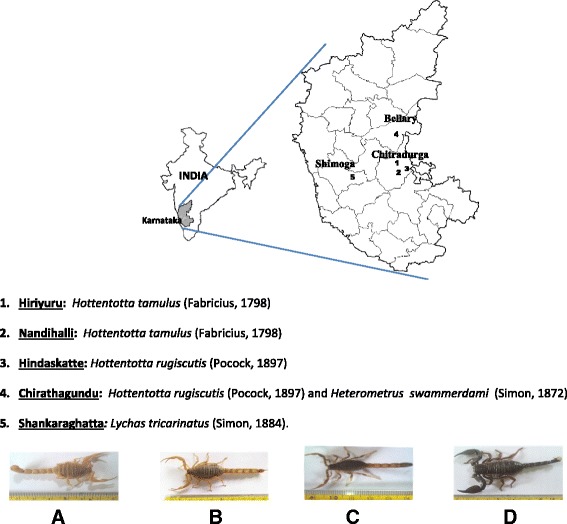
Map of Karnataka showing the locations of different scorpion species. At the bottom: **a** *Hottentotta rugiscutis* (Pocock, 1897); **b** *Hottentotta tamulus* (Fabricius, 1798); **c** *Lychas tricarinatus* (Simon, 1884)*,* **d** *Heterometrus swammerdami* (Simon, 1872)

The scorpions were collected from four different locations namely, Hiriyuru, Hindaskatte, Nandihalli (Chitradurga district) and Chirathagundu (Bellary district) of Karnataka, India (Fig. 1). Brown colored scorpions (*Hottentotta rugiscutis*, *Hottentotta tamulus* and *Lychas tricarinatus*) were found underneath the stone and wood materials over damp soil. Black scorpions (*Heterometrus swammerdami*) were found burrowed in soil at depths of 5–10 inches. These were captured carefully using long forceps by holding the tip of their tail without harming the animal. Black and brown scorpions were then transferred into separate containers with soil of about two inches in depth upon which small stones were placed. A maximum of 15 scorpions were kept in each container, which contained tiny holes for adequate ventilation.

### Identification of scorpions

Based on the morphological characteristics such as body color, the shape and size of pedipalps, shape of sternum, color of telson and number of pectine teeth, the collected scorpions were identified to the species level [10, 11].

### Maintenance of scorpions

**Fig. 2:**
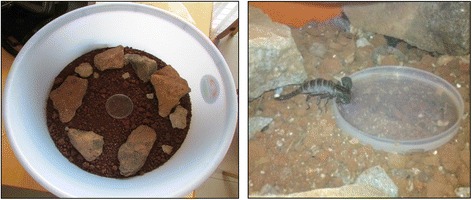
A scorpion habitat in the laboratory (left) and a black scorpion drinking the water (right). A plastic tub having soil substrate and stones mimicking scorpion habitat along with water-filled petri dish at the center as a water supplement. A black scorpion, *Heterometrus swammerdami*, drinking the water placed in the petri dish

In the laboratory, scorpions were placed in large plastic tubs (diameter: 54 cm; height: 22 cm) and cages [43 (l) × 27 (w) × 15 (h) cm] on top of a soil bed of about 3 inches in depth, above which proper size stones (~15 × 8 × 15 cm) were kept, mimicking their natural habitat. As a source of drinking water, a Petri dish lid containing tap water was kept at the center of the plastic tub/cage (Fig. 2). About 8–10 brown scorpions such as *Hottentotta rugiscutis* (Pocock, 1897)*, Hottentotta tamulus* (Fabricius, 1798), and the black scorpion, *Heterometrus swammerdami* (Simon, 1872), were housed in cages and plastic tubs respectively, and then covered with a mosquito net of proper dimensions.

Scorpions were fed once weekly such insects as grasshoppers, crickets and occasionally cockroaches [12]. The size of the insects fed to the scorpions was directly proportional to that of the scorpions themselves. At three-day intervals, the water content in the Petri dish was checked and, if necessary, refilled. Whenever necessary, soil was moistened with adequate water and unused dead insects and debris were removed. Scorpions found to be weak and mother scorpions with their young brood were housed in separate plastic containers to avoid any cannibalistic activity on the part of other scorpions. After 2 to 3 weeks, mother scorpions were placed in the common cage and scorplings were fed tiny insects. Molted scorpions were not fed insects for 3 to 4 days to avoid any physical damage to themselves, nor were subjected to venom extraction for about 20 days. The scorpions were manipulated only on the day of venom extraction and during the replacement of soil, which was carried out once every two months. The venom was not extracted on the day immediately after feeding and also no food was added until three days after the venom extraction [13, 14].

### Extraction of scorpion venom

**Fig. 3:**
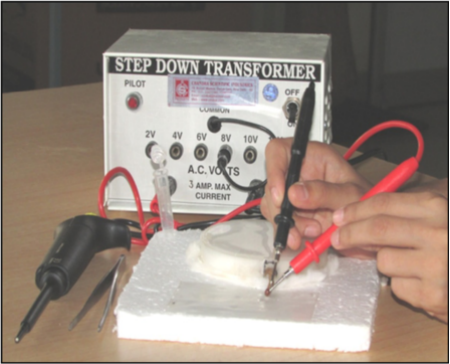
Extraction of venom by electrical stimulation method. Setup for milking of venom from *Hottentotta rugiscutis* by the electrical stimulation method using step-down transformer and restrainer

Extraction of venom was performed at monthly intervals by the electrical stimulation method using a restrainer developed by Deoras and Vad [15] with slight modifications. Scorpions were restrained over a rectangular shaped thermocol board (20 × 16 cm) affixed at the center with a thick layer of cotton. Above this, a Petri dish lid with cotton inside and an opening on its side sufficiently large for protrusion of the scorpion tail, was placed in an inverted position. The tail was held firmly using forceps and electrically stimulated by pointing two electrodes connected to a step-down transformer (Chandra Scientific Industries, India) at junctions between the tail segments, one next to the telson and the other at the junction between IV and V metasomal segment (Fig. 3). Specimens of *Hottentotta tamulus*, *Hottentotta rugiscutis* and *Heterometrus swammerdami* were stimulated twice at 8 V and 10 V respectively, for 2 to 3 seconds, with a short interval between two stimulations. The venom was allowed to secrete over a piece of parafilm placed at the base of its tail and then transferred to a vial. The venom obtained in a single episode from many scorpions of a species were pooled, mixed with excess double-distilled water and centrifuged at 15,000 rpm for 20 minutes to remove the mucus; the supernatant was lyophilized and stored at –20 °C until use. For protein estimation and toxicity studies, venom was resuspended in phosphate buffered saline, pH 7.0 [16, 17].

### Lethality of scorpion venom

Toxicity of venom from *Hottentotta rugiscutis* of Chirathagundu region was studied by estimating the LD_50_ value under *in vivo* conditions. Five different doses covering the range of mortality from 0 to 100 % were tested. The test group mice were subcutaneously injected with one of five concentrations (2.60, 2.80, 3.02, 3.26, 3.52 mg/kg b.w.) of venom dissolved in 0.2 mL of phosphate buffered saline (PBS). Control group was injected with equivalent volume of PBS only. The animals were monitored for 24 hours and the LD_50_ was calculated by the graphical method of Miller and Tainter [18].

## Results and discussion

A total of 351 brown scorpions and 22 black scorpions were collected at different time intervals from different regions of eastern Karnataka (Table 1). Transporting the scorpions in containers mimicking their natural habitat was apparently helpful in minimizing any sort of stress on them. Brown scorpions collected from the Hindaskatte and Chirathagundu regions were identified as *Hottentotta rugiscutis*, whereas the scorpions from Nandihalli and Hiriyuru were identified as *Hottentotta tamulus*. The black scorpion from Chirathagundu was identified as *Heterometrus swammerdami.* Heretofore, the scorpion species, *H. rugiscutis* has not been reported from any part of Karnataka [10]. Although this study was focused on the collection of scorpions from the regions previously stated, we also collected another scorpion species haphazardly from the premises of Kuvempu University, Shivamogga district, with the aim of displaying the diversity of scorpions in Karnataka; it was identified as *Lychas tricarinatus* (Simon, 1884), but maintenance and venom collection were not carried out for this species (Fig. 1).

**Table 1:** Numbers of six scorpion species collected from five different regions of Karnataka

Region	Chirathagundu		Hiriyuru	Nandihalli	Hindaskatte	Shankaraghatta
Species	*Hottentotta rugiscutis* (Pocock)	*Heterometrus swammerdami* (Simon)	*Hottentotta tamulus* (Fabricius)	*Hottentotta tamulus* (Fabricius)	*Hottentotta rugiscutis* (Pocock)	*Lychas tricarinatus* (Simon)
No. of scorpions	181	22	10	45	95	20

The scorpions maintained in cages and plastic tubs were active and healthy with a survival rate of 97 %. Employment of a Petri dish as a water reservoir makes water easily available to all scorpions (Fig. 2). This has proved to be a more efficient and easier method than the use of wet cotton balls placed in a container [13, 14]. Adequate spraying of water restores the moisture content of the soil bed and is also helpful in the molting of scorpions [19].

Feeding of scorpions once a week was noteworthy as it was observed that most of them did not consume insects when supplied at an interval of 3–4 days. It was crucial that insects be smaller than the scorpion to ensure that the latter can grab the prey easily with its pincers. All the insects were added to tubs/cages with their wings and forelimbs chopped so that they were easily accessible to the scorpions.

**Fig. 4:**
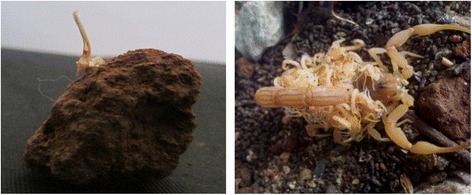
Spermatophore and mother scorpion with young brood. A spermatophore attached on the surface of a soil clod found in the container (left) and mother scorpion with its young brood which were delivered during captivity (right)

During the period of captivity, molting was observed at least twice in *Heterometrus swammerdami,* and only once in few *Hottentotta rugiscutis* and *Hottentotta tamulus*. Few scorpions gave birth to offspring, which may have been conceived after captivity, as we observed spermatophores (sperm containing tube-like structures) attached to mud particles or stones (Fig. 4) in many plastic tubs/cages, which is an indication of mating activity in laboratory conditions. Although it was not our motive to rear the scorplings, they were maintained on moral grounds, but ended up with only a 2 % survival rate. Rearing of scorplings in captivity is a difficult task with a much lower success rate as compared to the maintenance of adult scorpions [20], as also reported by Candido and Lucas and Gopalakrishnakone [14, 21]. As it was observed, such factors as the size and type of feed, moisture content of soil and temperature of the environment may affect the survival of these scorplings.

### Extraction of venom by electrical stimulation method

In total, 6.0 mL of venom was obtained from *Hottentotta rugiscutis* species at monthly intervals for the duration of 18 months (data not shown). The venom was also collected from other species for a short period in order to validate the stimulator and modified restrainer used. The *H. rugiscutis* and *H. tamulus* scorpions secreted a transparent watery pre-venom on initial stimulation, whereas on second stimulation, the venom obtained was viscous and whitish in color. But this secretion pattern was not consistently observed in all the scorpions of the particular species stimulated. However, *Heterometrus swammerdami* secreted relatively more viscous and whitish venom in all the stimulations. The quantity of venom procured from an individual scorpion varied with its size. The average amount of venom secreted from each individual scorpion in a single extraction procedure was ~ 8 μL during the period of first 12 months, but reduced to ~ 5 μL per scorpion for the next six months. Thus, maintaining the scorpions for more than 18 months may not be sufficiently productive in the context of venom yield, which was also noted by Candido and Lucas [14]. *H. swammerdami* secretes venom which is less toxic as they are relatively large in size and possess strong pedipalps, thus enabling them to attack the prey without the need of highly toxic venom. In contrast, *H. rugiscutis* and *H. tamulus* scorpions, which are small in size and possess relatively weak pedipalps, produce a more highly toxic venom [22]. *H. rugiscutis* is responsible for many envenomations in the region of Chirathagundu village. In an extensive survey of the literature, we found hardly any reports on the lethality of this species. In this context, further studies were carried out only on *H. rugiscutis* venom.

The stimulator used in the present study for the extraction of venom was more affordable than the one mentioned by Lowe and Farrell [8] and can be employed in the field if connected to a small battery. The voltage required to stimulate the scorpions varies according to species and size. The venom from the species *Androctonus mauretanicus* and *Buthus occitanus tunetanus* were extracted by stimulating at 12 V, whereas *Parabuthus* sp.*, Tityus serrulatus and Heterometrus gravimanus* were stimulated at 40 V, 12.5 V and 8 V, respectively [9, 13, 14, 16]. The present study also revealed similar variations. *H. rugiscutis* and *H. tamulus* secreted the venom when stimulated at 8 V, while the *H. swammerdami* secreted the venom only at 10 or 12 V based on species size. The expression time reported in this study (2–3 s) was shorter than the finding of 15 s by Gopalakrishnakone *et al*. [21]. These variations can also be due to different stimulators and conditions used in those studies.

The restrainer used herein was simple, cheap and effective, which eliminated any possibility of injury to the animal or the handler. The spontaneous expulsion of venom is a concern that was avoided by anesthetizing the scorpion with 5 % CO_2_ or by placing it in a refrigerator (0 °C) for 3 to 5 minutes prior to venom collection [13, 16]. However, during this study, proper handling and use of the modified restrainer drastically reduced the problem of spontaneous expulsion of venom. Although the use of thermocol in the restrainer does not cause any problem, in the future it should be replaced with a wooden base to which a petri dish can be fixed with a spring holder to enable an easy restraining process.

### Toxicity of scorpion venom

The protein content of venom from *Hottentotta rugiscutis* and *H. tamulus* were 0.79 mg and 0.70 mg/mg of lyophilized venom. The only report found until the present on the lethality of *H. rugiscutis* scorpion venom records an LD_50_ value of 4.2 mg/kg of body weight via intraperitoneal route [23], but no report was found on the amount of protein present in that venom. In this study, the LD_50_ of this venom was estimated to be 3.02 mg/kg of body weight via subcutaneous route. Lethality of *H. rugiscutis* scorpion venom was determined using different doses after which the death or survival ratio was recorded over a 24-hour period (Table 2). The symptoms observed in the experimental animals after venom injection included agitation, hyper-excitability, sweating, paralysis, salivation, squeaking, hunched back movements, convulsions, weakness and death.

**Table 2:** LD_50_ of *H. rugiscutis* venom injected to mice subcutaneously (*n* = 5) and calculated by graphical method of Miller and Tainter

Group	Dose (mg/kg bw)	Log of dose	Volume of injection (mL)	Mice (death/total)	Percentage of death (%)	Corrected percentage	Probit value
1.	2.60	0.414	0.2	0/5	0	5^a^	3.36
2.	2.80	0.447	0.2	1/5	20	20	4.16
3.	3.02	0.480	0.2	3/5	60	60	5.25
4.	3.26	0.513	0.2	4/5	80	80	5.84
5.	3.52	0.544	0.2	5/5	100	100^a^	6.64

^a^Correction formula: $0\%=100\left(\frac{0.25}{n}\right)100\%=100\left(n-0.25/5\right)$; bw: body weight

## Conclusions

The method followed in this study for scorpion maintenance in the laboratory is valuable. The modified restrainer and stimulator are significantly efficient in venom extraction for both smaller and larger scorpions. This was the first report for the presence of *H. rugiscutis* in Karnataka. Furthermore, the elevated population of *H. rugiscutis* in the Chirathagundu region presents a threat to human life that requires the development of a therapeutic anti-venom.

## Ethics committee approval

All the experiments were performed according to the guidelines approved by the Institutional Animal Ethics Committee (National College of Pharmacy, Shivamogga, Karnataka; no: NCP/IAEC/CL/206/01).
